# Molecular and Clinical Aspects of Targeting the VEGF Pathway in Tumors

**DOI:** 10.1155/2010/652320

**Published:** 2010-06-10

**Authors:** Grzegorz Korpanty, Laura A. Sullivan, Elizabeth Smyth, Desmond N. Carney, Rolf A. Brekken

**Affiliations:** ^1^Department of Medical Oncology, Mater Misericordiae University Hospital, Eccles St, Dublin 7, Ireland; ^2^Division of Surgical Oncology, Department of Surgery, Hamon Center for Therapeutic Oncology Research, University of Texas Southwestern Medical Center, Dallas, TX 75390, USA; ^3^Department of Pharmacology, University of Texas Southwestern Medical Center, Dallas, TX 75390, USA

## Abstract

Tumor angiogenesis is a complex process resulting from many signals from the tumor microenvironment. From preclinical animal models to clinical trials and practice, targeting tumors with antiangiogenic therapy remains an exciting area of study. Although many scientific advances have been achieved, leading to the development and clinical use of antiangiogenic drugs such as bevacizumab, sorafenib, and sunitinib, these therapies fall short of their anticipated benefits and leave many questions unanswered. Continued research into the complex signaling cascades that promote tumor angiogenesis may yield new targets or improve upon current therapies. In addition, the development of reliable tools to track tumor responses to antiangiogenic therapy will enable a better understanding of current therapeutic efficacy and may elucidate mechanisms to predict patient response to therapy.

## 1. Introduction

Angiogenesis, the formation of new blood vessels from a pre-existing vascular network, is a crucial process during tumor development. New vessels are needed to supply the tumor with nutrients for sustained local growth and to enable distant metastases [1]. The idea of tumors promoting their own angiogenesis through the secretion of then unknown factor(s) was first proposed in the 1930s by Gordon Ide [2]. In 1945, Glenn Algire [3] noticed that tumors grew significantly faster than normal tissues in part because of the ability to stimulate the growth of new vessels to provide oxygen and nutrients. In late 1960s, work by Bruce Warren, Melvin Greenblatt and Philippe Shubik [4, 5] supported the crucial role of tumor angiogenesis in malignant tumorigenesis. Their studies confirmed the hypothesis that tumors secrete soluble substances that promote vessel formation. Folkman reported the isolation of such a substance from a Walker 256 carcinoma grown in rats and called it a “tumor angiogenic factor” (TAF). In 1971, Folkman proposed that tumors cannot grow beyond a certain size without inducing angiogenesis and proposed that inhibiting tumor angiogenesis could prevent local tumor growth and formation of distant metastases [6, 7]. Since then extensive research has focused on the identification of proangiogenic factors produced by tumor cells and strategies to block their action. 

In 2004, bevacizumab (Avastin, Genentech, Inc.) became the first antiangiogenic drug approved by the Food and Drug Administration (FDA) for use in patients with metastatic colorectal cancer [8]. Since then bevacizumab has been approved for several other tumor types including breast, renal cell carcinoma, nonsmall cell lung cancer (NSCLC), and glioblastoma. Additionally, other antiangiogenic drugs were developed, such as sunitinib malate (Sutent, Pfizer, Inc.) and sorafenib tosylate (Nexavar, Bayer Pharmaceuticals Corp.), which are FDA approved for renal cell carcinoma and for gastrointestinal stromal tumors and hepatocellular carcinoma, respectively. The results of the antiangiogenic therapy in clinic have been disappointing as compared to the promising data from preclinical animal studies. Therefore, there is much to be learned about tumor angiogenesis and how best to utilize antiangiogenic therapy. In this review we will discuss the mechanisms of tumor angiogenesis and clinical application of antiangiogenic therapy.

## 2. Molecular Mechanisms of Tumor Angiogenesis

Growing tumors secrete a number of growth factors that can induce angiogenesis. One predominant factor that stimulates tumor angiogenesis is vascular endothelial growth factor A (VEGF). VEGF was initially identified as Vascular Permeability Factor (VPF) in 1983 by Harold Dvorak and Donald Senger in the conditioned medium of a guinea pig cancer cell line [9]. In 1989, Napoleone Ferrara's group reported the isolation and sequencing of an endothelial cell specific mitogen from pituitary cells and called it VEGF [10]. In the same issue of Science, Daniel T. Connolly reported cloning a gene encoding VPF that turned out to be identical with VEGF [11].

VEGF stimulates proliferation and migration of vascular endothelial cells (ECs). It also promotes survival, inhibits apoptosis, and regulates permeability of ECs. VEGF belongs to a family of growth factors that includes VEGF-B, -C, -D, -E, and placental growth factor (PlGF) [12]. Alternative splicing of the VEGF gene results in formation of four major isoforms of VEGF of varying molecular weights (VEGF_121_, VEGF_165_, VEGF_189_, and VEGF_206_). The main difference between these isoforms is bioavailability of VEGF for receptor binding. VEGF_121_ exists as a highly soluble circulating form while VEGF_206_ remains bound exclusively to the extracellular matrix (ECM) and is released upon proteolytic cleavage by metalloproteinases (MMPs) or plasmin [13]. VEGF_165_ is the predominantly active isoform that can be found both circulating in plasma and bound to ECM [12]. 

The biological functions of VEGF are mediated upon binding to receptor tyrosine kinases Vascular Endothelial Growth Factor Receptor-1, -2 (VEGFR1, 2). VEGFR1 binds VEGF, VEGF-B, and PlGF [14, 15]. VEGFR1 participates in embryonic vessel development, and is proposed to facilitate hematopoiesis and recruitment of endothelial cell progenitors to tumor blood vessels from bone marrow [12]. VEGFR1 binds VEGF with high affinity (*K*_*D*_ ~ 10–20 pM), which is significantly stronger than the affinity of VEGF-VEGFR2 interaction. The mechanisms through which VEGFR1 functions have not been elucidated completely. Genetic data suggest that during embryonic development VEGFR1 functions as a negative regulator of VEGF activity. Mice deficient in VEGFR1 (e.g., *VEGFR1^−/−^*) die *in utero* from an over abundance of endothelial cells that are disorganized [16]. Furthermore, mice engineered to express VEGFR1 lacking the tyrosine kinase domain (f*lt-1^TK-/-^*) develop normally with a fully functional vascular network [16]. Additionally, the extent of VEGFR1 involvement in adult angiogenesis is not well-defined [17, 18]. There are numerous reports of selective blockade of VEGFR2 activity in tumors resulting in reduced angiogenesis and tumor growth [19–23] suggesting that VEGFR1 activity is not required for VEGF-induced angiogenesis in pathological conditions. However, the function of VEGFR2 is defined more clearly.

**Figure 1:**
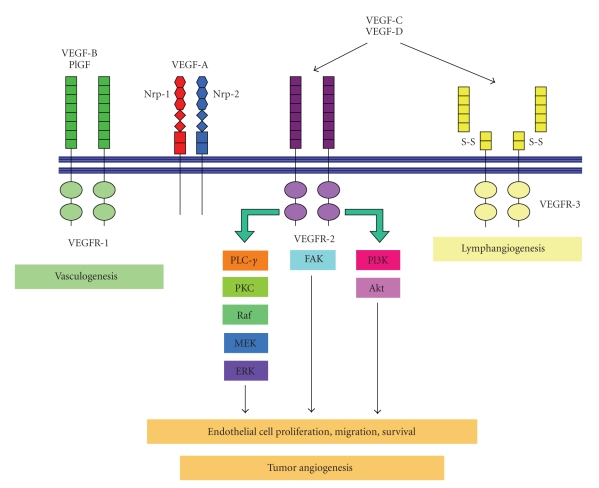
VEGF signaling interactions. The VEGF family can bind to VEGFR1, VEGFR2, and VEGFR3 inducing signaling cascades to promote vasculogenesis, angiogenesis, and lymphangiogenesis, respectively.

VEGFR2 is the key mediator of VEGF-driven angiogenesis. VEGFR2 is crucial during embryonic vascular development. Heterozygous and homozygous VEGFR-2 knockout mice die *in utero* due to disrupted vasculogenesis and hematopoiesis [24]. Upon VEGF binding, VEGFR2 undergoes auto-transphosphorylation and downstream effectors including phospholipase C gamma, protein kinase C, Raf, the MAP kinase signaling cascades, and the PI3K and FAK pathways are activated, leading to endothelial cell proliferation, migration, and survival (Figure 1) [25, 26]. VEGFR3 binds VEGF-C and -D and is directly involved in formation of the lymphatic vasculature physiologic and tumor development [27, 28]. There is also experimental evidence that VEGFR3 mediated activation of lymphatic endothelial cells is crucial for metastasis [29]. Neuropilin-1 (Nrp-1) and Neuropilin-2 (Nrp-2) are coreceptors originally identified for their involvement in neuronal guidance, and that bind members of collapsin/semaphorin protein family [30]. The Nrps can also bind to certain heparin binding isofoms of VEGF (e.g., VEGF_165_) to enhance the binding of VEGF to VEGFR1, and VEGFR2 (Figure 1) [31, 32]. Nrps lack tyrosine kinase domains but do contain an intracellular PDZ domain, which has been suggested to facilitate VEGF specific signaling. 

VEGF expression within tumors is regulated by oxygen levels, growth factors and cytokines, and oncogene activation/tumor suppressor inactivation [26]. Hypoxia in the tumor microenvironment is one of the most important factors influencing expression of VEGF. Hypoxia inducible factor-1 (HIF-1) is a transcription factor that regulates expression of certain genes in response to intracellular oxygen levels [33, 34]. It consists of two subunits: alpha (*α*) and beta (*β*). Normoxic conditions favor ubiquitin-dependent proteosome-mediated degradation of HIF-1*α* subunit, while oxygen deprivation stabilizes and enhances HIF-1*α*/HIF-1*β* dimerization. These dimers interact with a hypoxia response element (HRE) in the promoter region of many genes, including VEGF [35–37]. VEGF expression is also regulated via paracrine or autocrine release of growth factors and cytokines such as platelet-derived growth factor (PDGF), epidermal growth factor (EGF), keratinocyte growth factor, insulin-like growth factor (IGF), transforming growth factors alpha and beta (TGF-*α*, -*β*), interleukin 1*α* and 6 (IL-1*α*, -6) and prostaglandins (PGE2) [38–43]. During tumorigenesis, certain genetic mutations in the ras oncogene or Wnt-signaling pathways may also lead to elevated expression of VEGF [44, 45]. Tumor-derived VEGF may also function in an autocrine manner [46]. Receptors for VEGF (e.g., VEGFR1, VEGF2, Nrp1, Nrp2) are expressed on multiple cancer cell lines [47, 48], and there is evidence that VEGF can function as a cell survival factor for tumor cells and vascular endothelial cells within the tumor [49, 50].

The idea of vascular progenitor cells derived from bone marrow that incorporate into the tumor vasculature is exciting and controversial [51]. Circulating VEGF as well as other growth factors produced by tumor can mobilize variety of hematopoietic cell populations that express CD45, VEGFR1, VEGFR2, VE-cadherin, tie-2 or CXC chemokine receptor 4 [52–56]. There is a significant discrepancy among the published studies regarding the percentage contribution of bone marrow-derived cells into the formation of tumor vasculature—numbers vary between as high as 50% to as low as 5% [52, 57–59]. However, Robert Kerbel's group observed that after exposure to chemotherapy or vascular disrupting agents (VDAs), there is a significant efflux of circulating bone marrow-derived cells (BMDC) homing to the sites of tumor vasculature [60, 61]. This phenomenon may have a potent clinical application if confirmed in human studies. Using agents that can block incorporation of BMDCs may contribute to better outcomes of chemotherapy by interfering with tumor angiogenesis.

## 3. Clinical Applications of Antiangiogenic Therapy

The VEGF pathway can be targeted therapeutically at various molecular levels. Currently two major concepts are studied in the clinical setting: blocking VEGF from binding to its extracellular receptors with VEGF antagonists (antibodies, VEGF-Trap) or inhibiting VEGF signaling with tyrosine kinase inhibitors (TKIs) [62]. As previously mentioned, bevacizumab is a humanized, VEGF-neutralizing antibody that was the first antiangiogenic agent approved by the FDA for use in cancer patients. In 2004, a pivotal phase III clinical trial demonstrated a 4.6 months survival benefit of adding bevacizumab to chemotherapy in patients with metastatic colorectal cancer [8]. After the encouraging data from this trial were published, patients with other solid malignancies were enrolled into a multitude of clinical trials that added bevacizumab into the standard treatment of care. However, the results from many of these clinical trials are disappointing. Most patients fail to achieve long-term benefits with bevacizumab plus chemotherapy [63]. Selected groups of patients respond with tumor shrinkage, disease stabilization, or improvements in survival that are counted in months rather than years [64].

A new approach to anti-VEGF therapy currently being evaluated is genetically engineered fusion proteins that function as molecular “traps” for VEGF. Aflibercept (VEGF-Trap, Regeneron Pharmaceuticals, Inc.) is a recombinant fusion protein that binds both VEGF and PlGF with high affinity. It is composed of the extracellular domains of VEGFR1 and VEGFR2 that are fused to the Fc region of human IgG [65]. Currently, there are more than 40 ongoing trials (http://clinicaltrials.gov/) that explore this therapy in solid and hematologic malignancies.

**Figure 2:**
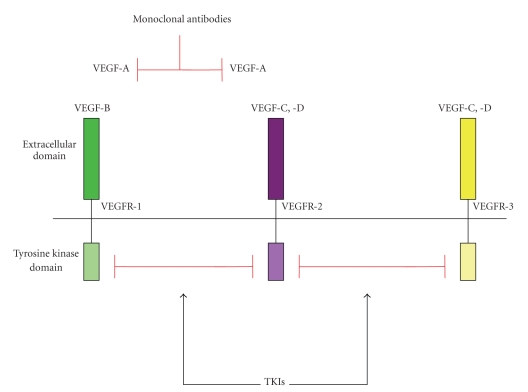
Inhibition of VEGF signaling pathways. Several classes of drugs have been developed to combat VEGF-mediated tumor angiogenesis. Monoclonal anti-VEGF antibodies (e.g., bevacizumab) and soluble receptor constructs (e.g., VEGF-Trap) bind to the VEGF and PlGF preventing their interaction and signaling through VEGFR1 and VEGFR2. Tyrosine kinase inhibitors (TKIs) inhibit the intracellular tyrosine kinase activity of VEGF receptors, blocking downstream signaling.

Small molecule TKIs with antiangiogenic activity are another important area of active clinical research. Unlike monoclonal antibodies (i.e., bevacizumab) or fusion proteins (i.e., aflibercept), TKIs are small molecules that interfere directly with tyrosine kinase activity (Figure 2). Since the intracellular domain targeted by TKIs is structurally similar in many tyrosine kinase receptors, a single TKI usually interferes with the activity of multiple receptors [66]. Sunitinib and sorafenib are multitargeting TKIs that can block activity angiogenic targets such as of VEGFR1, 2, 3, platelet-derived growth factor (PDGF) receptors and c-Kit or RET. After confirmed clinical benefit for sunitinib and sorafenib in selected patient groups [67, 68], there are now a variety of ongoing clinical trials recruiting patients from a broad spectrum of solid malignancies (http://www.clinicaltrials.gov/).

## 4. Mechanisms of Action of Antiangiogenic Agents

Various agents that target tumor angiogenesis are currently under investigation in different cancer types in many clinical trials [62]. While some of these agents show more encouraging results than the others, a common clinical problem is the lack of effective tools to monitor tumor response to these novel therapies [69]. The *Response Evaluation Criteria in Solid Tumors* (RECIST) criteria that are commonly used to monitor tumor response may not be an effective or even accurate measure of response to antiangiogenic agents. As an example, antiangiogenic agents will often enhance the central necrosis of tumors without changing the overall tumor size, which is a central parameter in RECIST evaluation [70].

An area of intense debate is how antiangiogenic agents actually work in terms of combating cancer [71]. According to the Folkman hypothesis, interference with tumor angiogenesis results in either inhibition of new vessel formation or progressive loss of existing vessels supporting tumor growth. An inadequate blood supply caused by a reduction of the vascular network in response to antiangiogenic therapy, slows and eventually prevents tumor growth and causes the tumor to regress to a “state of dormancy”, which can be clinically undetectable [7]. Evidence for this paradigm can be found in preclinical studies where fast-growing human tumors are treated with anti-VEGF therapy for long periods of time [72, 73]. 

An alternative explanation for anti-VEGF activity and possibly antiangiogenic agents in general is anchored in the heterogeneity of tumor vasculature. A minority of tumor blood vessels are associated intimately with pericytes and as a result are more functional and stable [74]. These vessels are not as dependent on VEGF stimulation for survival. In contrast, a large proportion of tumor blood vessels are tortuous, leaky, and immature, lacking interactions with pericytes. Furthermore, these vessels are more dependent on survival signals provided by VEGF and other growth factors. When VEGF levels are decreased via therapy these vessels regress, leaving behind a more stable vascular network. There is also compelling evidence that VEGF actively suppresses pericyte recruitment, therefore blocking VEGF activity may also result in the active recruitment of pericytes to remaining blood vessels [75]. As a result, the vasculature that remains in the face of anti-VEGF therapy consists of a higher percentage of pericyte associated blood vessels that are more efficient in function. This process has been termed “normalization” by Jain who hypothesizes that anti-VEGF therapy actually “normalizes” tumor vasculature and transiently improves blood flow within the tumor, thus enhancing the delivery of chemotherapy [76, 77]. Additionally, because stable vessels within the tumor are less leaky, interstitial pressure may decrease and thereby facilitate tissue penetration of chemotherapy. A supportive corollary to this is that antiangiogenic therapy has been shown to increase the efficacy of radiation therapy due to transient improvement in tumor oxygenation as a result of antiangiogenic treatment and vascular normalization [71, 78]. 

## 5. Monitoring Clinical Response to Antiangiogenic Therapies

The majority of noninvasive techniques used to assess the effects of antiangiogenic therapy do not directly visualize tumor blood vessels. Rather surrogate markers for vascular function such as blood flow are used commonly. These techniques rely on the fact that during the course of treatment blood flow within the tumor changes, either increasing due to normalization or decreasing due to diminished blood supply and vessel regression [79–81]. Hemodynamic changes within the tumor vasculature remain the major surrogate markers for majority of these techniques. Clinically relevant imaging techniques include magnetic resonance imaging (MRI), computed tomography (CT), positron emission tomography (PET), and ultrasound (US). Each of these techniques can be used with appropriate contrast media to evaluate hemodynamic function within tissues including solid tumors. 

Perfusion dynamic contrast-enhanced (DCE) MRI has been used successfully in both preclinical and clinical models to follow hemodynamic function [82]. DCE-MRI makes use of paramagnetic tracers, mostly consisting of a low-molecular-weight gadolinium (Gd) and is the standard method for measurement of vascular function in clinical trials of antiangiogenic drugs [83]. Signal enhancement obtained by DCE-MRI depends on tissue perfusion and permeability, contrast concentration, and extravascular space volume [84]. DCE-MRI has been especially useful in clinical studies of patients with liver and brain tumors [85–90], and has been investigated as a possible pharmacodynamic biomarker sorafenib therapy in metastatic renal carcinoma [91].

CT-based perfusion imaging techniques are also used to assess the vascular effects of antiangiogenic treatments [92, 93]. Although DCE-MRI gives better spatial resolution and is a superior method for brain imaging studies, CT still remains a preferred method for imaging structures within the thorax, abdomen, and pelvis. Thus some clinical studies investigating antiangiogenic agents have used perfusion CT rather than DCE-MRI to evaluate tumor blood flow [94–96].

In addition, PET-based imaging techniques are widely used in clinical oncology [97]. PET uses positron-emitting tracers, of which H_2_^15^O can be used to study tumor blood flow and this method has been used in clinical trials with good results [98]. H_2_^15^O is a positron-emitting tracer that can diffuse freely into the tissues and its tissue uptake correlates with blood perfusion [99]. Both H_2_^15^O PET and DCE-MRI are useful for monitoring tumor microvasculature. H_2_^15^O PET is particularly useful in the assessment of tissue perfusion while DCE-MRI measures also vascular permeability. A major disadvantage of both methods is their limited availability for patients because they require highly skilled and trained staff, that is, typically only available in large radiology or nuclear medicine departments.

Worldwide, ultrasound (US) is one of the most commonly used noninvasive imaging techniques. It provides anatomical information and can also be used to assess physiological function (e.g., blood flow with doppler ultrasound) or to serve as a therapeutic tool (e.g., high frequency ultrasound ablation of the tissue) [100, 101]. Because blood is only slightly less echogenic than surrounding tissue, US is not very effective for imaging small blood vessels. However, the introduction of US contrast agents expanded the clinical and research applications of US especially in the area of vascular imaging. Microbubbles (MB) are small particles (1–10 *μ*m) consisting of a gaseous core and a shell of protein (e.g., albumin) or lipid mixture [102] that can be injected intravenously and are promising US contrast agents. MBs are intravascular tracers that do not extravasate unless there is structural damage to the vessel wall. When injected intravenously, MBs enhance the echogenicity of the blood pool and enable distinction of vascular structures from the surrounding tissue. Within in an ultrasound field MB resonate in response to the ultrasound wave and can enhance both grey scale images and flow mediated doppler signals. Their high echogenic properties are due to the difference of compressibility of the gaseous core within the MB and the surrounding blood components and tissue [103]. MB have proven their usefulness in clinical echocardiography, especially in the evaluation of systolic myocardial function, ejection fraction, delineating endocardial border, and myocardial blood flow [104–106]. Imaging metastatic deposits or primary liver tumors (e.g., hepatocellular carcinoma) with contrast US is an example of the clinical application for MB-enhanced US imaging [107, 108]. The liver is one of the organs, that is, most commonly affected by distant metastases, and early detection of small (subcentimeter) lesions by contrast-enhanced US is of clinical significance [109–111]. Comparative studies of the sensitivity and specificity of PET, CT, DCE-MRI, and MB-enhanced US for detection of tumor perfusion showed that contrast US is an effective and correlative method with significant clinical potential [112, 113].

MB behave hemodynamically like red blood cells, circulate freely after injection and are small enough to reach the capillary microcirculation [114]. The idea of targeted imaging using contrast US is based on the selective accumulation of MB in specific vascular beds that can be reached by US wave and subsequently imaged. MB with an albumin-containing shell can adhere to endothelial cells that are activated by inflammatory cytokines, or activated leukocytes, which enables MB to be targeted passively to the areas of vascular inflammation [115–117]. MB can be also targeted actively to specific vascular beds by conjugation of targeting moieties (e.g., antibodies or peptides) to the MB shell [118–120]. In preclinical studies, MB have been targeted to various endothelial markers expressed on inflamed or ischemic tissues such as the myocardium or kidney [121–123]. Although tumor endothelial cells are often thought to be genetically normal, work by Hida et al. has demonstrated that mouse endothelial cells harvested from tumor xenografts are aneuploid and have abnormal centromeres [124]. Perhaps related to this cytogenetic abnormality, tumor endothelial cells express specific molecules that are absent or expressed at a much lower levels on endothelium in normal noncancerous tissue. Thus, the tumor vasculature is an attractive subject for imaging with targeted MB and US [125, 126]. The list of potential target molecules selective for tumor vasculature is growing and includes growth factor receptors, integrins, ephrins, endoglin, tumor endothelial markers (TEMs), and markers of cell stress (see Table 1 at supplementary material available at 10.1155/2010/652320).

The development of surrogate markers of pathological angiogenesis to monitor the response of patients to antiangiogenic therapy is of critical importance if antiangiogenic strategies are to be a viable modality for cancer therapy. Contrast US using targeted MB can be an efficient tool to monitor the expression of surface markers by tumor endothelial cells. This strategy can be used to visualize tumor blood vessels and in addition can follow the expression level of markers that are known to be altered by antiangiogenic therapy. VEGFR2 is a commonly used marker of vascular endothelial cells and has been used by multiple groups as a molecular target for MB. Animal models of angiosarcoma, glioma, and breast cancer showed that VEGFR2 targeted MB enhanced US imaging in evaluation of tumor angiogenesis [127, 128]. Recently, our group evaluated vascular response to antiangiogenic and chemotherapy in mouse models of pancreatic cancer using MB targeted against VEGFR2, the VEGF:VEGFR complex, and endoglin [20]. Using three different formulations of tumor vessel specific MB and US, we were able to noninvasively monitor vascular function of subcutaneous and orthotopic pancreatic tumors in mice. We found that targeting to VEGFR2, endoglin, or the VEGF:VEGFR complex was specific for tumor vasculature as there was no signal enhancement in nontumor tumor tissue. Further, we found that anti-VEGF therapy or treatment with gemcitabine reduced the expression of the molecular targets bound by targeted MB. Our contrast US intensity data correlated with immunohistochemical analysis of tumor samples, providing the first indication that targeted MB could be used to follow expression of a cell surface target. Additionally, these studies also validated that gemcitabine can effect endothelial cells in tumors. Other groups have since confirmed our findings using targeted MB to image the response of tumor vessels to the therapy [129]. These data and the work of others [130–133] that conclusively demonstrate the utility of contrast US using targeted MB support the clinical evaluation of such strategies as a method for following response to antiangiogenic therapy in cancer patients.

## 6. Toxicities of Antiangiogenic Therapies

VEGF signaling is involved in many normal physiologic processes such as hemostasis, vascular homeostasis and integrity, and the maintenance of endothelial function in kidney glomeruli [134]. Following the introduction of bevacizumab into the clinic, toxic side effects became apparent. The most common side effects of bevacizumab and other antiangiogenic agents are hypertension (3–36 % of patients) and proteinuria (21–64 % of patients) [135]. Although the exact pathophysiological mechanism is not yet fully understood, there is evidence coming from both animal and clinical models, that bevacizumab increases the risk of renal thrombotic microangiopathy [136]. It has been shown in animal models that after binding to VEGF, bevacizumab-VEGF immune complexes can be deposited in the glomerular basement membrane contributing to the development of both proteinuria and hypertension [137]. Bevacizumab has also been shown to increase the incidence of hemorrhagic and thrombotic events in cancer patients. One of the most serious side effects observed in lung cancer patients are hemoptysis and pulmonary hemorrhage. In a phase II clinical trial, NSCLC patients with squamous histology were at a higher risk of developing fatal bleeding, that was most likely related to tumor necrosis and proximity of tumor to the large vessels [138]. Based on this observation bevacizumab is not recommended for squamous NSCLC. In addition, bevacizumab is not recommended for patients with pre-existing conditions that may predispose for either thrombotic or hemorrhagic events (e.g., brain metastases). Bevacizumab can also potentiate the incidence of side effects that are specific to chemotherapy treatment like neutropenia, infections, and thrombocytopenia [139]. There have also been reports of potentially serious toxicities such as nasal septum perforation, reversible posterior leukoencephalopathy syndrome (severe hypertension, cortical blindness, and seizures) or osteonecrosis of the jaw, although these events are very rare [140–142]. TKIs have a unique toxicity profile and are more commonly associated with rash due to blocking EGFR activity and gastrointestinal symptoms like nausea, diarrhea due to the administration of the drug. Hypertension is the predominate toxicity associated with sorafenib and sunitinib treatment due to their antiangiogenic specificities [143]. Endothelial cell production of nitric oxide and prostacyclin is required for mediating vasodilatation and controlling blood pressure. These mediators are stimulated by VEGF-induced VEGFR2 signaling, which is blocked by sorafenib and sunitinib treatment. Further, TKIs have recently been reported to increase patient risk of bleeding events due to interruption of VEGF-mediated vascular homeostasis [144].

## 7. Future Directions

Despite the modest survival benefits observed in clinical practice, antiangiogenic therapy remains an attractive concept. Only five years have passed since the first antiangiogenic drug was approved by the FDA for a clinical use. Although we have learned important lessons about this new class of cancer drugs, many questions still remain. The multitude of ongoing clinical trials testing both the new agents and different combinations of agents with already established clinical benefits, may shed light on multiple questions regarding antiangiogenic therapy [145]. Better selection of patients, including the therapeutic schedule of antiangiogenic therapy (i.e., adjuvant versus palliative), monitoring of clinical response, and better toxicity profile of antiangiogenic drugs are among the most important clinical aspects that the ongoing clinical trials will address. In addition, intrinsic and acquired resistance of tumors to antiangiogenic therapies is a growing concern in the clinic, as most patients fail to show sustained benefit with continuous therapy [146]. There are many possible mechanisms of resistance to antiangiogenic therapy that are being actively investigated. One possibility is the activation of alternative molecular pathways resulting in ongoing angiogenesis in response to the presence of a selective inhibitor (e.g., fibroblast growth factor (FGF), interleukin-8, ephrins, angiopoietins, SDF-1 pathway activation, and increased VEGF expression following epidermal growth factor blockade) [147–149]. Also, resistance has been linked to an unresponsiveness of pericyte-covered tumor vessels to antiangiogenic therapies [150, 151], and to the hypovascularity and low levels of *de novo *angiogenesis characteristic of some tumors (e.g., pancreatic cancer) [152]. Although antiangiogenic therapies have entered into clinical practice, we still lack a reliable marker(s) of treatment efficacy. Studies on noninvasive marker(s) such as blood levels of circulating growth factors, cytokines and/or endothelial progenitor cells gave mixed results and are not validated at the present time for a clinical use [153]. Future studies that involve complex proteomic-based analysis may help to find a noninvasive way to not only monitor the effects by also to better select patients who may benefit from antiangiogenic therapies.

## Supplementary Material

Table 1 summarizes potential target molecules selective for tumor vasculature identified so far.
