# Supplementary material for: Molecular and Clinical Aspects of Targeting the VEGF Pathway in Tumors
Source: J Oncol. 2010 Jun 10;2010:652320. doi: 10.1155/2010/652320 (PMC2902148; doi:10.1155/2010/652320)
Supplement: Supplementary file 1 — Table 1 summarizes potential target molecules selective for tumor vasculature identified so far. [file 652320.f1.pdf]

Table 1. Potential markers of tumor blood vessels

Targeting moiety	Antigen	Location of marker	Reference
Multiple	VEGF:VEGFR	Angiogenic BV	[154] [155] [156] [157]
MKID2	$\alpha_3\beta_1$	Angiogenic BV	[158]
GoH3	$\alpha_6\beta_1$	Angiogenic BV	[158]
EN7/44	p30.5	Proliferating EC	[159]
Multiple	CD105 (endoglin)	Proliferating EC	[160] [161] [162] [163] [164] [165]
FB5	Endosialin	Proliferating EC	[166]
MK 2.7	VCAM-1	Activated EC	[167]
	E-selectin, CD62E	Activated EC	[167]
4A11	H-5-2, Lewis^{y}-6	Activated EC	[168]
	CD44	Activated EC	[169]
Metastatin	Hyaluronan	Activated EC	[170]
Vitaxin; RGD cyclic peptide	$\alpha_V\beta_3$; $\alpha_V\beta_5$	Activated EC	[171] [172] [173]
Multiple	$\alpha_1\beta_1$; $\alpha_2\beta_1$	Activated EC	[174] [175]
Multiple	$\alpha_5\beta_1$	Activated EC	[176]
3SB, 3G4	Phosphatidylserine	Activated EC	[167]
TV-1	FN	Basement membrane	[177]
L19	ED-B isoform of FN	Basement membrane	[178] [179] [180] [181]
HUIV26, HUI77	Denaturated collagens	Proteolyzed basement membrane	[182] [183]
Multiple	NG2 proteoglycan	Pericytes	[184]
NGR peptide	CD13/APN	Tumor EC	[185]

(Abbreviations used: APN, aminopeptidase N; BV, blood vessels; EC, endothelial cells; FN, fibronectin; MMP, matrix metalloproteinases; PSMA, prostate specific membrane antigen; SMC, smooth muscle cell; TEM, tumor endothelial marker; VEGF:VEGFR, complex of VEGF and its receptor.)
